# ‘A foreigner is not a person in this country’: xenophobia and the informal sector in South Africa’s secondary cities

**DOI:** 10.1186/s42854-022-00046-4

**Published:** 2023-01-19

**Authors:** Godfrey Tawodzera, Jonathan Crush

**Affiliations:** 1 University of the Western Cape, Cape Town, South Africa; 2 Balsillie School of International Affairs, Waterloo, Ontario Canada

**Keywords:** Xenophobia, Informal sector, Violence, Migrants, South Africa

## Abstract

South Africa’s major cities are periodically wracked by large-scale xenophobic violence directed at migrants and refugees from other countries. Informal sector businesses and their migrant owners and employees are particularly vulnerable targets during these attacks. Migrant-owned businesses are also targeted on a regular basis in smaller-scale looting and destruction of property. There is now a large literature on the characteristics and causes of xenophobic violence and attitudes in South Africa, most of it based on quantitative and qualitative research in the country’s major metropolitan areas. One of the consequences of big-city xenophobia has been a search for alternative markets and safer spaces by migrants, including relocating to the country’s many smaller urban centres. The question addressed in this paper is whether they are welcomed in these cities and towns or subject to the same kinds of victimization as in large cities. This paper is the first to systematically examine this question by focusing on a group of towns in Limpopo Province and the experiences of migrants in the informal sector there. Through survey evidence and in-depth interviews and focus groups with migrant and South African vendors, the paper demonstrates that xenophobia is also pervasive in these smaller centres, in ways that both echo and differ from that in the large cities. The findings in this paper have broader significance for other countries attempting to deal with the rise of xenophobia.

## Science highlights

- Xenophobic prejudice and violence against migrant informal sector enterprises in South Africa could spread elsewhere on the continent.
- Migrants move to secondary centres in an effort to reduce the risk of harm but xenophobia is also growing in these smaller centres.
- There are differences between xenophobia in major cities and secondary centres in relation to vulnerability to crime and to police abuse of power.

## Policy & Practice Recommendations

- Xenophobia is not restricted to large cities and mitigation policies also need to target secondary urban centres
- Service delivery protests in South Africa lead to opportunistic xenophobic attacks on migrant-owned businesses. Addressing the root causes of protests would reduce the vulnerability of migrants.
- Xenophobic violence directed at informal sector operators could be reproduced in other African cities unless proactive policies are adopted by governments.

## Introduction

Rapid urbanization, the expansion of urban food deserts, and general food system transformation in recent decades have been accompanied by major expansion of the informal food sector in African cities (Crush et al., 2017; Holdsworth and Landais 2019; Skinner and Watson, 2020). Despite intense competition from supermarkets, informal vendors play a central role in making food available and accessible to households in low-income neighbourhoods in most primary and secondary cities and towns (Blekking et al. 2020, Crush and Frayne 2011; Skinner, 2019). In some countries, the sector has also provided important niche opportunities for migrants and refugees excluded from formal employment opportunities to establish and grow food-related enterprises, particularly in urban food markets, informal settlements and inner-city areas (Bosiakoh and Tetteh 2019; Chinyemba 2017; Macharia and Van den Broeck, 2016; Young and Crush, 2020). South Africa is the continent’s largest recipient of migrants from elsewhere in Africa and here, too, they have a visible foothold in the informal food sector of major cities such as Cape Town (Battersby et al. 2016; Rogerson 2018) and Johannesburg (IOM 2021, Peberdy 2021). One recent study, for example, found that 50% of enterprises in Cape Town’s informal food sector were run by migrants to South Africa despite the operating challenges posed by xenophobic violence and official apathy (Tawodzera 2019).

Xenophobic attitudes and actions have traditionally been associated with citizen hostility towards immigrants and refugees in Europe and the United States (Favell and Baumgartl 1995; Fetzer 2000; Lee 2019; Yakushko 2018). However, there is growing evidence for the rise of xenophobia in many African countries as well (Abidde and Matambo 2021; Akinola 2018; Mafukata 2021; Moyo and Mpofu 2020; Nyamnjoh 2014). South Africa is often singled out in this literature as the country in which xenophobic sentiments and violence are at their most pervasive, intense and destructive. The rise of xenophobia accompanying the collapse of apartheid and new forms of migration to South Africa is well-documented (Crush, 2001; Harris 2001; HRW 1998, 2020). South Africa’s major cities are now periodically roiled by widespread xenophobic attacks on migrants and refugees. In the worst instance of xenophobic violence in 2008, sporadic attacks on migrants and refugees descended into two weeks of nationwide hayhem as armed mobs destroyed homes and property owned by migrants, murdered over 70 people, seriously injured thousands, and hounded over 100,000 migrants out of their communities (Crush 2008; Hassim et al. 2008). While the South African state has steadfastly refused to admit that xenophobia is a driving force or even present in the country, others have sought to recast the issue as one of anti-African sentiment (Afrophobia) rather than xenophobia against migrants in general (Crush 2022, Tewolde 2010).

Since 2008, xenophobic violence has increasingly been directed at migrant small business owners and their employees running food and other enterprises in the urban informal sector (Crush and Ramachandran 2015; Ramachandran et al. 2017; Tevera 2013). South Africa lacks the large formal and informal urban food markets characteristic of the rest of Africa, so the protections offered to migrant food vendors by clustering in safe spaces are generally absent. Isolated and vulnerable, migrant-owned businesses on the country’s “mean streets” have become targets of xenophobic violence, destroying livelihoods and increasing food insecurity in major cities for migrants and their customers in the process (Crush et al. 2015, 2017). Migrants operate in a continual climate of fear and uncertainty since there is often little or no warning of impending violence. The government position is that opportunistic criminality rather than xenophobia is the motivator of violence against migrant enterprises (Gordon 2019; Hiropoulos 2020).

A logical extension of this official explanation is that South African-owned informal businesses are equally at risk and experience similar rates of robbery, looting, destruction of property and physical violence. This argument has indeed been advanced by some researchers (Charman and Piper 2012; Piper and Charman 2016; Piper and Yu 2016). However, it has also been undermined by other researchers who view xenophobia as the driving force in selective discrimination and targeting (Gastrow 2013, 2022; Moyo 2017; Tengeh 2016; Cinini and Mkhize 2021). The question of whether violence and its causes in the urban informal sector is scale-dependent has not been explored, Most of the evidence comes from research conducted in the country’s large cities rather than in smaller, secondary urban centres. Indeed, this is a characteristic of the xenophobia literature writ large which depends for its evidence on data from the major cities and not secondary centres.

This paper focuses on the phenomenon of xenophobia in smaller South African centres and seeks to answer three inter-related questions. The first is whether international migrants establish informal food and other enterprises in secondary centres in South Africa and, if so, who they are and their reasons for moving to these centres. The second question emerges from the first and asks whether migrants and South Africans running informal businesses are equally vulnerable to violence or whether the former are more likely to be targeted and, if so, what is the explanation. Finally, if it can be established that xenophobia is wholly or partially a factor, as it is in the large cities, then it is important to assess whether it takes the same form in secondary urban centres and with the same consequences or differs from the experience in the major metropolitan areas. In this paper, we address all three questions using quantitative and qualitative data from our research in six small towns in the province of Limpopo, some 300 km north of Johannesburg, close to the South African border with Zimbabwe.

## Methodology

We were able to identify only two previous published studies of relevance conducted in Limpopo Province, neither of which was based on a large sample or conducted in more than one centre. Mothibi et al. (2015), for example, interviewed only 18 migrant enterprises and no South Africans in the town of Louis Trichardt. Kgaphola et al. (2019) also focus on one area – Mahkweng – where they surveyed 50 mainly migrant food businesses (known as spazas). The research for this paper therefore differed from other studies by surveying a representative sample of over 1000 informal business owners in several different towns, using a mixed quantitative and qualitative methodology, and sampling both international and South African migrant owned enterprises.

The survey of informal sector operators was conducted in secondary South African urban centres in Limpopo Province in 2016. The primary aim was to sample an equal number of South African and migrant informal sector enterprise-owners (500 of each) operating in the same localities. Without a census or register of informal sector businesses to create a sampling frame, an alternative strategy was used to ensure a degree of representativeness of the two samples. Three procedures were adopted: first, maximum variation sampling (MVS) was used to identify a sub-set of towns in which to conduct the research. MVS is based on the principle of maximum diversity, an extension of the statistical principle of regression. Six towns, covering a wide size range and scattered around the province, were selected ranging in size from 130,000 to less than 10,000 (Table 1).

**Table 1:** Study sites

	2011 Census Population Size	Migrant Sample	%	South African Sample	%
Polokwane	130,028	159	31.6	166	29.3
Thohoyandou	69,453	59	11.7	57	10.1
Musina	42,678	121	24.1	74	13.1
Louis Trichardt	25,360	36	7.1	51	9.0
Tzaneen	14,571	33	6.5	177	31.3
Burgersfort	6369	96	19.0	41	7.2
	288,459	504	100.0	566	100.0

A field audit identified the approximate size of the informal sector in each urban centre. The centres were allocated a sample proportional to the size of the sector as a whole. Within each centre, the same systematic sampling approach was adopted. Sampling was conducted along street lines in each site. The mapped grid-pattern exhibited by streets was utilized, sampling one street after the other in successive fashion moving from west to east. After identifying the first five enterprises on a street, and randomly selecting the first of the five for interview, every third enterprise was selected thereafter. Surveys were administered to migrant respondents who were either born in another country (international migrants) or outside the centre within South Africa (internal migrants). Where business owners were not available for interview, field workers made three call backs to the enterprise, after which a substitution was made. The total number of migrant and South African entrepreneurs interviewed in each site is shown in Table 1. For this paper, the 504 migrant responses and 566 South African responses from the six centres were aggregated to form two comparable data sets. Table 2 shows the country of origin of the migrant sample.

**Table 2:** National origins of migrant survey sample in Limpopo

	No.	%
SADC Countries		
Zimbabwe	56	11.1
DRC	55	10.9
Angola	3	0.6
Mozambique	3	0.6
Lesotho	1	0.2
Tanzania	1	0.2
Zambia	1	0.2
Sub-Total	120	23.8
Other Africa		
Ethiopia	141	28.0
Somalia	37	7.4
Ghana	34	6.8
Nigeria	29	5.8
Eritrea	23	4.6
Sudan	5	1.0
Cameroon	4	0.8
Burundi	3	0.6
Congo Brazzaville	3	0.6
Kenya	1	0.2
Rwanda	1	0.2
Uganda	1	0.2
Sub-Total	282	56.2
Asia		
Bangladesh	49	9.7
Pakistan	27	5.4
India	4	0.8
China	3	0.6
Sub-Total	83	16.5
Other	18	3.5
Total	503	100.0

To supplement the quantitative survey data, a total of 26 in-depth interviews were conducted with migrant vendors from various countries about their experiences since arriving in South Africa. These interviews were supplemented with four focus groups with 6–10 migrants from a single or two countries in each group.  

## Results and discussion

In this section of the paper, we present findings from the survey administered to the sample of informal enterprise operators. The first question concerns whether migrants have a presence in the informal sector of secondary urban centres and, if so, where they originate. The international migrant cohort proved to be extremely heterogeneous originating from seven other countries in the Southern African region (24%), twelve countries elsewhere in Africa (56%), and four Asian countries (17%) (Table 2). The largest number were from Ethiopia, followed by Zimbabwe, the DRC and Somalia. Migrants from Bangladesh, India, Pakistan and China were interspersed with those from African countries.

**Fig. 1:**
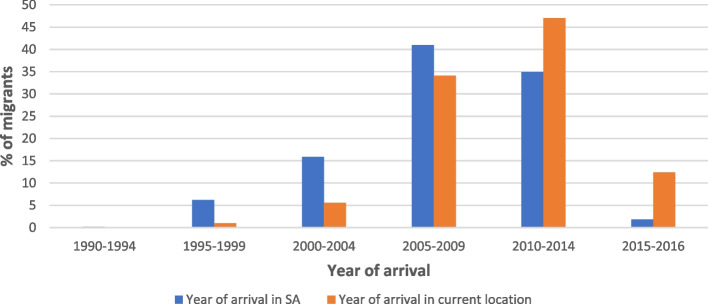
Timeline of arrival in South Africa and Limpopo Province

Most of the migrants had moved to South Africa relatively recently. Only one had come before 1994 (Fig. 1). As many as 41% came between 2005 and 2009 and another 35% between 2010 and 2014. Figure. 1 shows that there was a general time lag between the date of arrival in South Africa and the date of moving to their Limpopo location. While 22% came to South Africa before 2005, only 7% arrived in one of the secondary urban centres in Limpopo prior to that date. Again, while 63% arrived in South Africa before 2010, only 42% had been living in a Limpopo town from before that date. This means that many did not come directly to Limpopo towns but relocated there from other urban centres.

Nearly two-thirds of the sample (64%) had lived in at least one other urban centre prior to moving to their current location. Table 3 summarises the urban centres in which migrants previously resided. Johannesburg was easily the most important initial destination with nearly one-third of the migrants having lived there. Some migrants had also lived in other major South African cities including Pretoria (13%), Durban (5%) and more distant Cape Town (5%). As many as 16% of the sample had moved from another urban centre within Limpopo Province to their current location and 20% had lived in secondary centres in other South African provinces. This indicates a general pattern of diffusion out from an initial larger centre down the urban hierarchy to secondary centres to set up or run an informal enterprise.

**Table 3:** Urban centres of previous residence

	No.	% of total
Large Cities		
Johannesburg	148	29.4
Pretoria	65	12.9
Durban	25	4.8
Cape Town	23	4.6
Port Elizabeth	12	2.4
Bloemfontein	3	0.6
Secondary Urban Centres		
Other Limpopo	78	15.5
Other	101	20.1

*Note*: Multiple response question

The second research question is whether international migrants and South Africans in the informal sector face the same challenges and have the same negative experiences. If they do, explanations other than xenophobia would need to be sought for any evidence of targeting of international migrants. Table 4 shows both similarities and differences between the two groups. As many as 30% of migrants reported conflict with South African business-owners, but so did 21% of South Africans. Again, while 22% of South Africans reported conflict with migrants, 26% of migrants reported conflict within their own group. Thus, both groups had very similar levels of conflict with each other and within their group. This suggests that conflict between migrants and South Africans is not necessarily motivated purely by xenophobia. The other similarity between the two groups lies is that both were victims of theft of goods and stock. While 38% of migrant businesses had been victimized, so had 31% of South Africans. While xenophobia cannot be discounted as a motive for robbery of migrants, this finding suggests that both groups are roughly equal victims.

**Table 4:** Challenges faced by informal business-owners

	International Migrants % Yes	South Africans % Yes
Levels of Conflict		
Conflict with foreign-owned businesses	25.6	22.1
Conflict with South African-owned businesses	30.2	21.4
Incidence of Theft		
Theft of goods/stock	38.3	30.4
Theft of money/income	31.3	11.8
Levels of Xenophobic Prejudice		
Verbal insults against my business	35.3	6.9
Prejudice against my nationality	47.6	1.1
Xenophobic Violence		
Physical attacks/assaults by South Africans	19.0	1.6
Police Misconduct		
Harassment/demands for bribes by police	26.2	3.9
Confiscation of goods by police	18.8	4.9
Arrest/detention	9.1	1.2
Physical attacks/assaults by police	8.7	1.1
N	504	566

There the similarities between migrants and South Africans end. International migrants are far more likely to be victims of theft of business income (31%) than South Africans (12%) for example. An even starker difference is evident when it comes to levels of prejudice with migrants far more likely to suffer verbal abuse against their business and prejudice on the basis of their nationality. Nearly half of all migrants reported the latter. Finally, very few of the South Africans reported that they had been victims of police misconduct, in contrast to migrants who reported more demands for bribes by the police (26% versus 4% of migrants), greater police confiscation of goods (19% and 5%) and more physical assaults (9% and 1%).

The third question addressed in this section is whether the experiences of migrants differ between secondary urban centres and large cities. To answer this question, we compared the challenges faced by informal business owners in Limpopo and Cape Town drawing on data from a companion survey of migrants in the informal sector in that city. Table 5 shows that the levels of within group and between group conflict and xenophobic prejudice are slightly higher in Cape Town while levels of xenophobic prejudice are very similar (experienced by 48% of migrants in both localities). There are two major differences between the two sites: first, in Cape Town the incidence of theft of both stock and income is significantly higher than in Limpopo. Second, the prevalence of police misconduct is reportedly higher in Limpopo than in Cape Town across all indicators.

**Table 5:** Challenges faced by migrants in Limpopo and Cape Town

	Limpopo % Yes	Cape Town % Yes
Levels of Conflict		
Conflict with foreign-owned businesses	25.6	28.3
Conflict with South African-owned businesses	30.2	34.9
Incidence of Theft		
Theft of goods/stock	38.3	56.7
Theft of money/income	31.3	44.2
Levels of Xenophobic Prejudice		
Verbal insults against my business	35.3	32.6
Prejudice against my nationality	47.6	48.0
Xenophobic Violence		
Physical attacks/assaults by South Africans	19.0	23.5
Police Misconduct		
Harassment/demands for bribes by police	26.2	10.7
Confiscation of goods by police	18.8	10.2
Arrest/detention	9.1	7.6
Physical attacks/assaults by police	8.7	6.8
N	504	500

The survey findings suggest that xenophobia is not only a feature of life in large South African cities but poses very real challenges to migrants seeking to build their enterprises in secondary centres as well. Migrant business-owners reported much higher levels of prejudice and verbal insult against their operations than South Africans. And nearly 20% of migrants surveyed in the six towns had experienced violent attacks on their businesses. By contrast, only 2% of the control group of South African internal migrant business-owners had been victims of violence. Victimization by the police was also a much bigger problem for migrants than their South African counterparts which also suggests that they were being singled out because they were foreign. At the same time, the xenophobic threat to migrant business operations was compounded by certain challenges common to all informal enterprises, local and foreign, especially theft of goods and stock.

The in-depth interview and focus group discussions shed additional light on the nature of the xenophobic challenge facing migrants in South African secondary centres. The survey findings show that many migrants in Limpopo moved there from the large cities. Since there is evidence that this is a general pattern across South Africa, the obvious question is why they do so (Crush and Ramachandran 2015). Since all of those surveyed run small informal businesses, the move could be a purely business decision motivated by new opportunities and markets and/or a response to the greater competition that exists in the informal sector in major cities. However, the in-depth interviews and focus groups elicited many examples of migrants moving from larger to smaller urban centres to escape xenophobic violence, often after they had been traumatised. Some of the respondents had been caught up in the nation-wide xenophobic violence that swept through large cities in 2008 and again in 2015. Others had been victims of attacks on their informal businesses in large cities including in Johannesburg, Port Elizabeth and Pretoria:

“Many times, my business was robbed when I was in Johannesburg. It was because I was a foreigner because they rarely stole from the locals. Sometimes criminals would come to you and ask you to give them money and they would just ask you the foreigner. Why not the local people? That is xenophobia.”

“In 2007 I was in Port Elizabeth and seven thugs robbed me and I went to the police and told them the name of the tsotsis (thugs). The police just said “My brother, I don’t want to die for your safety”. I had a spaza shop and the criminals started breaking the shop and we reported to the police but the police said they don’t have enough vehicles at the moment so they said we must wait. We waited for them for 3 hours, and eventually after they arrived they started smoking my cigarettes and sipping my drinks. After they opened a case, they demanded money because they said they were doing me a favour.”

“When I was in Pretoria, I saw four spaza shops being burnt. They had goods worth over R4 million and nothing was recovered. These people who are at the forefront of xenophobia first break in and loot the shop, then they burn what is left so that you will not recover anything.”

“In Soweto (Johannesburg) I had seen two people being killed in broad daylight and they were all foreigners and their shops were robbed. So I wanted to go somewhere else. Orange Farm (south of Johannesburg) was a good area for business but it was not safe. As a foreigner you are always conscious of your security and you can feel that this place is not good. It is far from the Johannesburg CBD and there are few police there. I was robbed seven times in the period that I stayed in Orange Farm. There were many spaza shops around me, but they kept stealing from me. Is that not xenophobia? Why not steal from the locals? Most of the time the robbers would come at night and you are still operating. They pounce on you with sticks, spanners or iron bars and they hit you hard. So I was almost killed twice and I thought this is enough. Let me leave this place. Then I left and came here.”

Movement from one town to another in Limpopo could also be a response to xenophobic violence. One Eritrean migrant, for example, was hounded out of Burgersfort and moved to Musina, opting out of food selling in the process:

“I was running a spaza shop in a place called Quete. They robbed me at night and stole my goods and burnt the things that they could not carry. There were goods worth over R60,000 in the shop. They stole almost three quarters of the goods and burnt the goods that were left. So I lost more than R60 000. They almost killed me and I ran away. I came here with nothing. I am not going back there again. I came here because I was running for my life. I was not thinking of doing business, but of surviving. I was almost killed that night. Where I came here, friends took me in. For some time I was not doing anything because I did not have enough money. I had about R12 000 with me when I left Burgersfort. I used that money and some that I borrowed from friends to start a business here.”

A second issue illuminated by the in-depth interviews concerns the nature of xenophobic prejudice and verbal insult against migrant enterprise owners. As one respondent noted:

“If you are a foreigner you are always affected by xenophobia. There is no way that you can live here and not be affected. Xenophobia starts from your customer. Some customers are very rude and if you respond, they will talk to you in their language and scold you and then tell you to go back to your country. They have bad words for foreigners. I think there are very few people who have not been affected by xenophobia in this country. If you are a foreigner, it always affects you.”

Third, the interviews help to explain the puzzle in the survey findings that migrant and local informal enterprises are equally affected by theft of goods and stock, but migrants are twice as likely to be robbed of their business income. Unlike South Africans, most migrants are unable to open personal or business bank accounts and deposit their takings in a secure facility. Not only are they unable to access credit from the formal banking system to expand their operations, but it also means they have to keep their cash on the business premises or at home:

“The most pressing problem is that we cannot open bank accounts. The law says that we can open an account and we can work and study. But banks do not want to help us. It is as if they have their own laws that they use which are not the laws of the country. They do not even bother to give reasons for their refusal, they just refuse. They just tell you that you need a green ID book, but where do we get the green ID book? We are not South Africans.”

“Without a bank account, where does one keep his money? If you keep a lot of money with you in the shop or at home you are inviting criminals who will later come and rob you or even kill you. That is the problem that the government is creating for us. They should give us proper documents that will allow us to open accounts. Or at least compel the banks to allow us to open bank accounts with the current documents that we have.”

While the motives for robbery may not necessarily be xenophobic, migrants are more vulnerable to theft of business income because of well-documented institutionalized xenophobia within the banking sector (Lose et al. 2016; Tengeh 2013).

A fourth question relates to the finding that migrants are much more vulnerable to violent attack. While this suggests that migrant enterprises are victims of xenophobic targeting, the evidence is much stronger when the nature of many incidents of violence is considered. In the interviews and focus groups, it emerged that physical attacks and looting of business properties were often triggered by mass service delivery protests against the national, provincial and municipal governments. While the connection between protests against government service delivery and attacks on migrant-owned shops may not be obvious, it is clear from the accounts that migrant and not locally-owned businesses are targeted:

“In most cases, locals are protesting for water and electricity-service delivery. When they do that, some people take the chance in the confusion to steal. We are not the government. We do not provide water and we do not provide electricity. So why are we the target? It is because we are easy and they know that when our shops are looted, the police do not do anything. So we are just victims and the government fails to protect us from this irrational behaviour. Local shops open even during protests and they are very safe. You cannot understand why we are always the target.”

“When there is a protest the first victims are usually those whose shops will be open as the crowd can just get in and loot everything. However, sometimes you can also be a victim even when the shop is closed because when they cannot steal, they burn the shop just to spite you. It is bad, but that is what happens. One time a friend of mine watched his shop being burnt. There was nothing that he could do.”

This is certainly part of a more general pattern in South Africa where in the chaos and looting during anti-government demonstrations, migrant-owned rather than local businesses are targeted. The statement above that “when our shops are looted, the police do not do anything” compounds the situation and raises the general question of whether there is evidence of police complicity motivated by xenophobia within the ranks.

The finding above that police misconduct against migrants is more prevalent in Limpopo than in a large city such as Cape Town is amplified by the in-depth interviews. The primary complaint of survey respondents was their treatment at the hands of local police who they consistently maintained are corrupt, abusive and xenophobic. In 2013, the police in Limpopo launched a province-wide campaign to close migrant-owned businesses, dubbed Operation Hardstick. They closed down over 600 businesses, detained owners, confiscated stock, imposed fines and verbally abused the migrants. Migrant associations took the government to court and in a landmark judgment in 2014 won their case in the Supreme Court. The judgment against the provincial and national governments noted that the police actions “tell a story of the most naked form of xenophobic discrimination and the utter desperation experienced by the victims of that discrimination” (Supreme Court 2014). While the court’s judgment allowed the businesses to re-open, it did not stop other criminal action by the police as these extracts suggest: 

“We feel that we are not protected here in South Africa. It is if a foreigner is not a person in this country. We are attacked and injured or killed but nothing happens to the perpetrators. We report our cases to the police but they do nothing. Sometimes they come and talk to us and give us case numbers, but that is as far as it goes. We rarely hear of arrest, or if there is an arrest, the people, the criminals are released on bail and that is usually the end of it. There are times when we know the criminals and we tell the police, but nothing really happens to the criminals. We see them walking the streets and we are afraid of them.”

“All they want is to suppress us. It is as if their duty is to suppress us every day. That is what they do. The police are always looking for trouble. They are not here to help. Whenever they stop you, they are looking to find out what fault is there (with your shop). It is very difficult for them not to find something that is not right with you or your car or your stock.”

As well as a failure to protect, the police in these secondary centres reportedly prey on migrants by questioning documentation, demanding bribes and constantly harassing them:

“We do not have a good relationship with the police at all. They just want to take money from us. We know some of them by name and they come to the streets and take legally printed permits and they repossess them, pretending to go and check for authenticity, but they never return the documents unless you pay them. I know a person whose papers were taken and he had to pay to get them just this last week. It happens every day. If you walk in town today, you will see them. Today they are targeting Zimbabweans, Mozambicans and Malawians. We just ask that they should respect our legally issued papers. In some instances they ask you to pay R1,000, R1,500 or even R2,000. They do not ask you to pay directly, but they frustrate you until you pay.”

“Reporting cases actually creates problems for you. When you report that robbers have stolen R30 000 from you, it tells the police that you are making money and they will be your biggest problem. One of our biggest challenges is on bank accounts. Something must be done on bank accounts so that we do not become targets of criminals. When police stop you at a roadblock, they check the whole vehicle, even under your seats and they will be looking for money. Sometimes they even have knives and they can rip your car seats wanting to find money. The way they search us is humiliating. If they find you with money they will take some of it and you will have nothing to do. If you have R1000, they can take R500 and leave you to go. If you have R10 000, they can take even R5 000. You cannot refuse because they can shoot you. As a foreigner you should always have money in your pocket, that is your ID.”

“One of our challenges is the traffic police. They are a problem here. They come on most days demanding money, bribe money. When you give them, they also send their colleagues to demand more money and so it does not end. We do not even understand why the traffic police should come and harass us. We are not traffic and it is not their job to police us. They should be on the roads looking for traffic offenders, but they like coming here to us and asking for money. It is very bad and it is bad for business.”

## Conclusion

The survey revealed several distinctive features about the profile of migrants working in the informal sector in small-town Limpopo. First, despite the fact that Limpopo adjoins Zimbabwe, the major source of migrants to South Africa, the informal sector in these towns was peopled by an unexpectedly heterogenous group of business owners from many different African and Asian countries. Second, it was clear that most did not come directly to these towns on moving to South Africa. Instead, they lived and worked for varying lengths of time in major migrant destinations; that is, the large cities. Relocation to Limpopo was a decision that came later, partly to seek out new and less competitive markets, but also because of the trauma they had witnessed or experienced during attacks by large xenophobic mobs. In other words, large-city xenophobia has the effect of driving migrants down the urban hierarchy towards secondary centres which, at least initially and in principle, are safer spaces in which to start and grow an informal enterprise. If this was their hope and expectation, many were quickly disabused.

Xenophobia in small town Limpopo mirrors that in the large cities in several distinct ways, the primary difference being one of scale rather than substance. First, although the evidence is filtered through the narratives of migrants, xenophobic attitudes and hostility appear to be no less intense in secondary urban centres. While some have correctly argued that it is incorrect to typecast all South Africans as xenophobic (Tewold 2021), it seems that xenophobia itself is no respecter of scale. Second, xenophobic violence, when it occurs, is no less random, indiscriminate and destructive. The only difference appears to be that there is less loss of life involved in secondary centres. Third, as in the large cities, anti-government service delivery protests quickly spill over into mob attacks on informal businesses owned by migrants. Migrant narratives are clear that in the general mayhem, they and not South Africans running businesses in the same neighbourhood are the ones that are targeted. The targeting of migrant-owned businesses were confirmed by the surveys which, on most metrics, showed that migrants were more likely to be victims of violence, looting and theft. Fourth, even as xenophobic violence forced relocation from large cities to small towns, there were at least some accounts (one of which is quoted in this paper) of relocation within Limpopo to escape the violence and start afresh somewhere else.

This is the first study to systematically investigate the phenomenon of anti-migrant xenophobia in African secondary urban centres. The large and growing literature on xenophobia in post-apartheid South Africa draws for much of its evidence on case studies from the country’s large metropolitan areas. Studies of the drivers of xenophobic violence in the informal sector have similarly focused on the major cities where the largest outbreaks have occurred and where mob attacks are quickly reported by media outlets. The result of big-city bias is a general cloak of invisibility on the question of whether xenophobia and xenophobic violence also exists in South African secondary urban centres and, if so, are they different in any way from the situation in cities such as Cape Town and Johannesburg?

Because the informal sector is a major site of ongoing xenophobic violence in South Africa, we focused on whether migrants operating informal businesses in smaller secondary cities are able to operate without the fear and insecurity that plagues those operating in major urban centres. To understand the manifestations and experiences of xenophobia at the local level, we adopted a mixed methods approach, combining a representative survey of migrant-owned businesses in the study sites with in-depth interview and focus groups with migrants. We also surveyed a control group of South Africans working in the sector. This is important because there is a school of thought in South Africa that contends that South Africans are just as likely to be targeted as migrants and that attacks on the latter are wrongly typecast as xenophobic. The survey results conclusively demonstrate that there are significant differences between the experiences of the two groups which can only be attributed to xenophobia.

In the major cities, there is considerable evidence of police abuse of powers motivated by institutionalized xenophobia, including an unwillingness to arrest and prosecute violent offenders. While the police in secondary centres seem equally disinterested in bringing the perpetrators of xenophobic criminality to book, they do appear extremely committed to extortion from migrants. There is evidence of similar extortion and corruption in big cities but it appears to be particularly blatant in Limpopo where anyone in a uniform, including traffic police, get in on the act. Ordinary citizens know they are unlikely to fall foul of the law for criminal acts of xenophobia, while the supposed enforcers of the law act with even greater impunity. Migrants definitely believe, as one pointed out, that “nothing really happens” to xenophobic criminals inside and outside the state.

The findings in this paper have several policy implications. First, the South African Immigration Act of 2002 proposed a policy framework in which “xenophobia is prevented and countered both within Government and ciyil society.” This commitment has not been taken seriously with the result that xenophobia has festered and spread. The mitigation challenge is now that much greater than it was because, as the paper shows, xenophobic behaviour and violence is no longer confined to large metropolitan areas. Second, addressing other policy priorities – such as failing service delivery – would help to address xenophobic violence triggered by anti-government protests. Third, the emerging literature on xenophobia in the rest of Africa makes clear that unless other countries are more proactive in managing migration and clamping down on signs of xenophobia, South Africa could well be showing them a mirror of their own future.

The paper also has implications for future research on migration and xenophobia more generally. First, in South Africa at least, there are numerous small area studies with unrepresentative sample sizes and unsustainable generalizations. This paper demonstrates the wider value of adopting a mixed methodology and representative sampling in the study of xenophobic attitudes and actions. Second, this analysis confirms that much greater sensitivity is needed into the spatially uneven character of xenophobia within as well as between different countries. Finally, the connections between popular expressions of xenophobia, institutionalized xenophobia and state action and inaction need research attention. South Africa is a particularly glaring example of what happens when a state does not acknowledge the existence of xenophobia in society and the state itself. In other contexts, the connections may be more subtle and complex but they are still worthy of exploration.

## Data Availability

Survey instruments are available on request.
